# Involvement and Possible Role of Eosinophils in Asthma Exacerbation

**DOI:** 10.3389/fimmu.2018.02220

**Published:** 2018-09-28

**Authors:** Kazuyuki Nakagome, Makoto Nagata

**Affiliations:** ^1^Department of Respiratory Medicine, Saitama Medical University, Saitama, Japan; ^2^Allergy Center, Saitama Medical University, Saitama, Japan

**Keywords:** bronchial asthma, eosinophils, neutrophils, periostin, rhinovirus

## Abstract

Eosinophils are involved in the development of asthma exacerbation. Recent studies have suggested that sputum and blood eosinophil counts are important factors for predicting asthma exacerbation. In severe eosinophilic asthma, anti-interleukin (IL)-5 monoclonal antibody decreases blood eosinophil count and asthma exacerbation frequency. However, even in the absence of IL-5, eosinophilic airway inflammation can be sufficiently maintained by the T helper (Th) 2 network, which comprises a cascade of vascular cell adhesion molecule-1/CC chemokines/eosinophil growth factors, including granulocyte-macrophage colony-stimulating factor (GM-CSF). Periostin, an extracellular matrix protein and a biomarker of the Th2 immune response in asthma, directly activates eosinophils *in vitro*. A major cause of asthma exacerbation is viral infection, especially rhinovirus (RV) infection. The expression of intercellular adhesion molecule (ICAM)-1, a cellular receptor for the majority of RVs, on epithelial cells is increased after RV infection, and adhesion of eosinophils to ICAM-1 can upregulate the functions of eosinophils. The expressions of cysteinyl leukotrienes (cysLTs) and CXCL10 are upregulated in virus-induced asthma. CysLTs can directly provoke eosinophilic infiltration *in vivo* and activate eosinophils *in vitro*. Furthermore, eosinophils express the CXC chemokine receptor 3, and CXCL10 activates eosinophils *in vitro*. Both eosinophils and neutrophils contribute to the development of severe asthma or asthma exacerbation. IL-8, which is an important chemoattractant for neutrophils, is upregulated in some cases of severe asthma. Lipopolysaccharide (LPS), which induces IL-8 from epithelial cells, is also increased in the lower airways of corticosteroid-resistant asthma. IL-8 or LPS-stimulated neutrophils increase the transbasement membrane migration of eosinophils, even in the absence of chemoattractants for eosinophils. Therefore, eosinophils are likely to contribute to the development of asthma exacerbation through several mechanisms, including activation by Th2 cytokines, such as IL-5 or GM-CSF or by virus infection-related proteins, such as CXCL10, and interaction with other cells, such as neutrophils.

## Introduction

Bronchial asthma is a chronic disease with airway hyperresponsiveness (AHR), reversible airflow limitation, and airway inflammation (1, 2). Asthma is recognized as a heterogeneous disease that has different phenotypes with distinct clinical characteristics, or different endotypes with distinct functional or pathophysiological mechanisms including eosinophilic asthma or non-eosinophilic asthma (3, 4). Recent studies have suggested that eosinophils play important roles in the development of asthma exacerbation (5–7). Therefore, suppressing eosinophilic inflammation and distinguishing eosinophilic from non-eosinophilic asthma may be useful for the treatment or prevention of asthma exacerbation. In the present review, the involvement and possible role of eosinophils in asthma exacerbation is discussed.

## Ethics statement

Our studies in this review were approved by the Institutional Review Board of Saitama Medical University Hospital, and written informed consent was obtained from the patients.

## Role of eosinophilic inflammation in asthma

Eosinophils, which tend to accumulate at sites of allergic inflammation, contribute to the development of bronchial asthma. They release a number of mediators, including specific granule proteins, such as major basic protein (MBP), radical oxygen species, cytokines, such as granulocyte-macrophage colony-stimulating factor (GM-CSF) and interleukin (IL)-8, and lipid mediators, such as cysteinyl leukotrienes (cysLTs) (8, 9). However, previous studies investigating the effectiveness of anti-IL-5 monoclonal antibody (mAb) treatment for asthmatics have suggested that eosinophils may only play a small role (10, 11). For example, it has been reported that anti-IL-5 mAb reduces the sputum or blood eosinophil count, but has no effect on histamine-induced AHR or allergen-induced late asthmatic response (10), which suggests that eosinophils do not play a role in the development of AHR or allergen-induced airflow obstruction.

By contrast, the role of eosinophils in the development of airway remodeling has been established at a relatively early phase (12). Eosinophil-deficient mice are reportedly protected from peribronchiolar collagen deposition (13). Eosinophils produce transforming growth factor (TGF)-β (14), which may contribute to airway fibrosis. Additionally, eosinophils can produce cysLTs (15) and be a major cellular source of cysLTs in the airways of individuals with seasonal allergic asthma or aspirin-exacerbated respiratory disease (16, 17), which also contribute to airway remodeling. Anti-IL-5 mAb suppresses airway remodeling (reduction of tenascin, lumican, and procollagen III) as well as airway eosinophils expressing mRNA for TGF-β1 and concentrations of TGF-β1 in the bronchoalveolar lavage (BAL) fluid of asthmatics (18).

As for the role of eosinophils in asthma exacerbation, recent studies have reported that sputum and blood eosinophil counts are important factors for predicting asthma exacerbation (5, 6, 7). Treatment for normalizing sputum eosinophil counts can help prevent asthma exacerbation (5), and blood eosinophil counts are associated with exacerbation frequency (6, 7). Furthermore, in severe asthmatic patients with sustained blood or sputum eosinophilia, anti-IL-5 treatment decreases both blood eosinophil counts and asthma exacerbation frequency (19–21). Eosinophil-derived granule products, such as MBP mediate epithelial cell damage, thereby, inducing AHR (22, 23). In accordance with these findings, anti-IL-5 mAb is now prescribed for patients with severe eosinophil-dominant asthma in the clinical setting.

**Figure 1:**
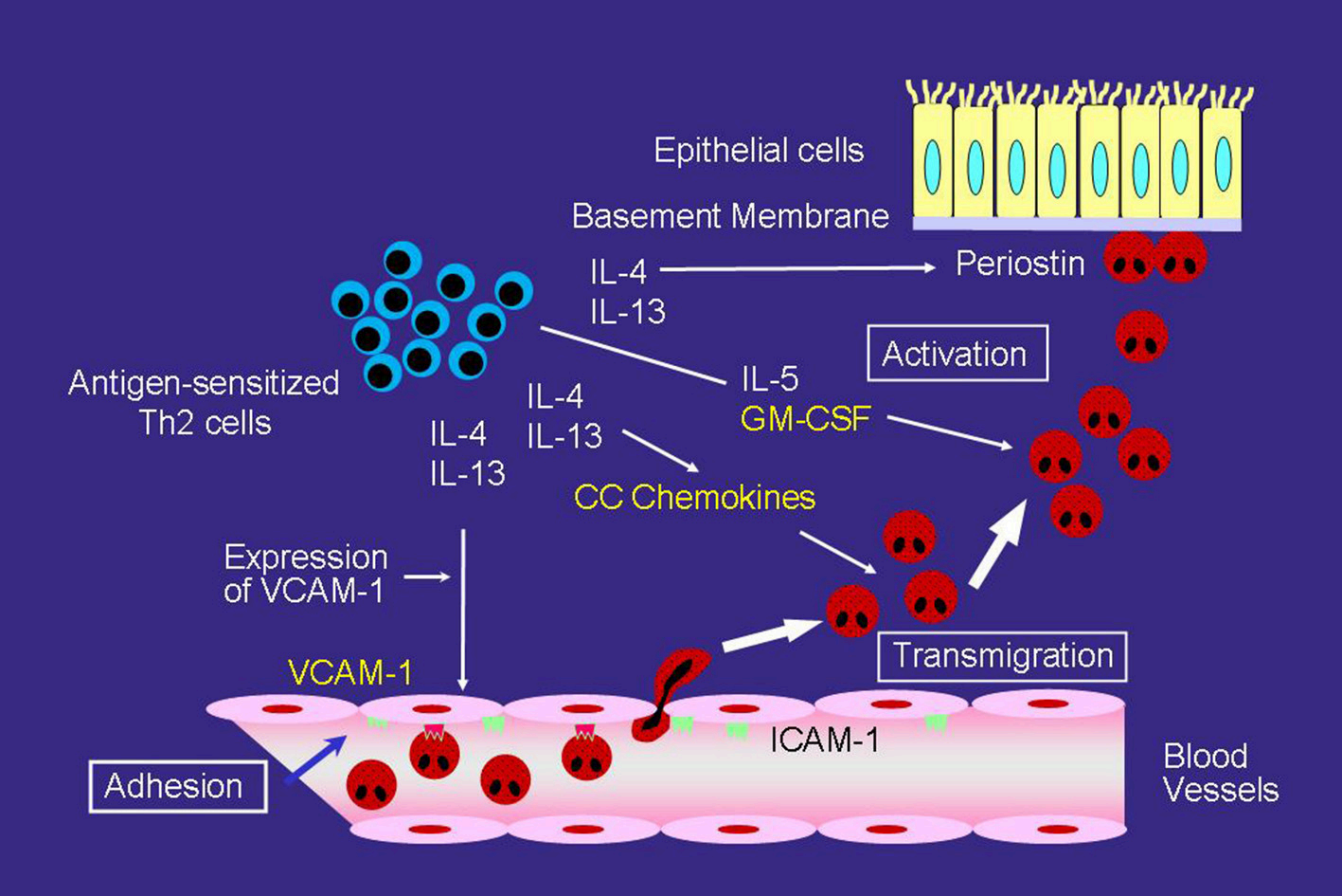
Mechanisms of eosinophilic airway inflammation in bronchial asthma. IL-5 plays an important role in the development of eosinophilic airway inflammation. However, in the absence of IL-5, the Th2 network, which includes a cascade of VCAM-1/CC chemokines/GM-CSF, can maintain eosinophilic infiltration and activation. Yellow text indicates a cascade of VCAM-1/CC chemokines/GM-CSF.

However, even with anti-IL-5 mAb, eosinophils can still accumulate and activate in the airways of asthmatics. One study reported that anti-IL-5 mAb may be insufficient to inhibit eosinophil activation in the airway (24). To accumulate in asthmatic airways, circulating eosinophils needs to adhere to vascular endothelial cells and then migrate over cells, which are regulated primarily by cytokines or chemokines induced by a number of cells, such as T helper (Th) 2 cells (Figure 1) (25). The crucial step for selective eosinophil recruitment is likely the adhesion of eosinophils with endothelial cells through the α4 integrin/vascular cell adhesion molecule (VCAM)-1 (25–27). The expression of VCAM-1 in endothelial cells is upregulated by IL-4 and IL-13, after which, blood eosinophils adhere spontaneously to VCAM-1 (28, 29). The interaction of eosinophils with VCAM-1 induces eosinophil superoxide anion (O${}_{2}^{-}$) generation and degranulation, and therefore may be the first step of eosinophil activation (28–30).

After adhering to the endothelial cells, CC chemokines, such as eotaxin and regulated upon activation, normal T-cell expressed and secreted (RANTES) effectively induce eosinophil transmigration over endothelial cells expressing VCAM-1 (Figure 1) (25, 31). A number of studies have reported an increase in CC chemokines in the airways of asthmatic patients. The airway expression of eotaxin and its receptor, CCR3, are elevated in atopic asthmatics compared with normal controls (32). In patients with acute eosinophilic pneumonia, monocyte chemotactic protein (MCP)-4, which is also a CCR3 ligand, is involved in the development of eosinophil transendothelial migration (33); however, the role of MCP-4 in asthma has yet to be fully clarified.

GM-CSF play an important role in eosinophil activation after migration process, even without IL-5 (Figure 1). GM-CSF induces eosinophil O${}_{2}^{-}$ generation and the release of specific granule proteins *in vitro* when incubated with VCAM-1 or intercellular cell adhesion molecule (ICAM)-1 (29). Furthermore, GM-CSF, but not IL-5, activates eosinophils of airways after segmental allergen challenge (34, 35). These findings suggest that in the absence of IL-5, the Th2 network, which includes a cascade of VCAM-1/CC chemokines/GM-CSF, is likely the primary pathway for maintaining eosinophilic infiltration and activation in asthma (25).

Moreover, cysLTs may be involved in the eosinophil accumulation in the airways of asthma. Inhalation of LTE_4_ stimulates the accumulation of eosinophils in asthmatic airways (36). LTD_4_ upregulates the β2 integrin expression of human eosinophils and increases eosinophil adhesiveness to ICAM-1 *in vitro* (37). Furthermore, β2 integrin-enhanced adhesion increases the effector functions of eosinophils. Therefore, cysLT-induced β2 integrin activation may be a key process in regard to cell activation in asthmatics (25, 29). In addition, LTD_4_ induces eosinophil transendothelial migration, O${}_{2}^{-}$ generation, and the release of specific granule proteins primarily through β2 integrin and the cysLT1 receptor (38). Furthermore, leukotriene receptor antagonist (LTRA) suppresses eosinophil airway inflammation *in vivo* (39–41). These findings suggest that cysLTs, along with the Th2 network, contribute to the development and maintenance of airway eosinophilic inflammation in asthma.

Periostin is an extracellular matrix protein that is highly expressed in the airways of asthmatics in response to Th2 cytokines, such as IL-13 (42), and is a biomarker of Th2-mediated immune responses in bronchial asthma (43, 44). Periostin also functions as a matricellular protein (42) that binds to cellular receptors and activates cells, including eosinophils. Periostin directly induces eosinophil adhesion, O${}_{2}^{-}$ generation, and degranulation through the αMβ2 integrin *in vitro* (45).

## Interactions of viral infection and eosinophils in the development of asthma exacerbation

Viral infection, especially rhinovirus (RV) infection, is a major cause of asthma exacerbation. In some community-based studies, viral infections have been identified in 80–85% of cases involving asthma exacerbation, and RV was found to be involved in about 65% of the patients in whom the causative virus was identified (46, 47). RVs have tremendous diversity (48). In addition to about 100 classical serotypes of the RV species A (RV-A) and B, over 60 types of RV-C were recently discovered by molecular techniques (48). Recent clinical data suggests that RV-C (49, 50) or RV-C and RV-A (51, 52) can induce more severe illness or asthma exacerbations, compared with other RVs, such as RV-B.

The numbers of not only neutrophils, but also eosinophils, increase in asthmatic airways during or after a viral infection (53–55). Experimental RV infection induces increased recruitment of eosinophils into the airway after segmental allergen challenge in allergic rhinitis patients, but not in non-allergic volunteers (53). Viral infection increases the eosinophil count in the airway epithelium of patients with allergic asthma (55), and high levels of eosinophilic cationic protein are observed in the sputum of asthmatic patients with viral infection (54). Therefore, eosinophils are indeed recruited to and activated in asthmatic airways during or after a viral infection.

**Figure 2:**
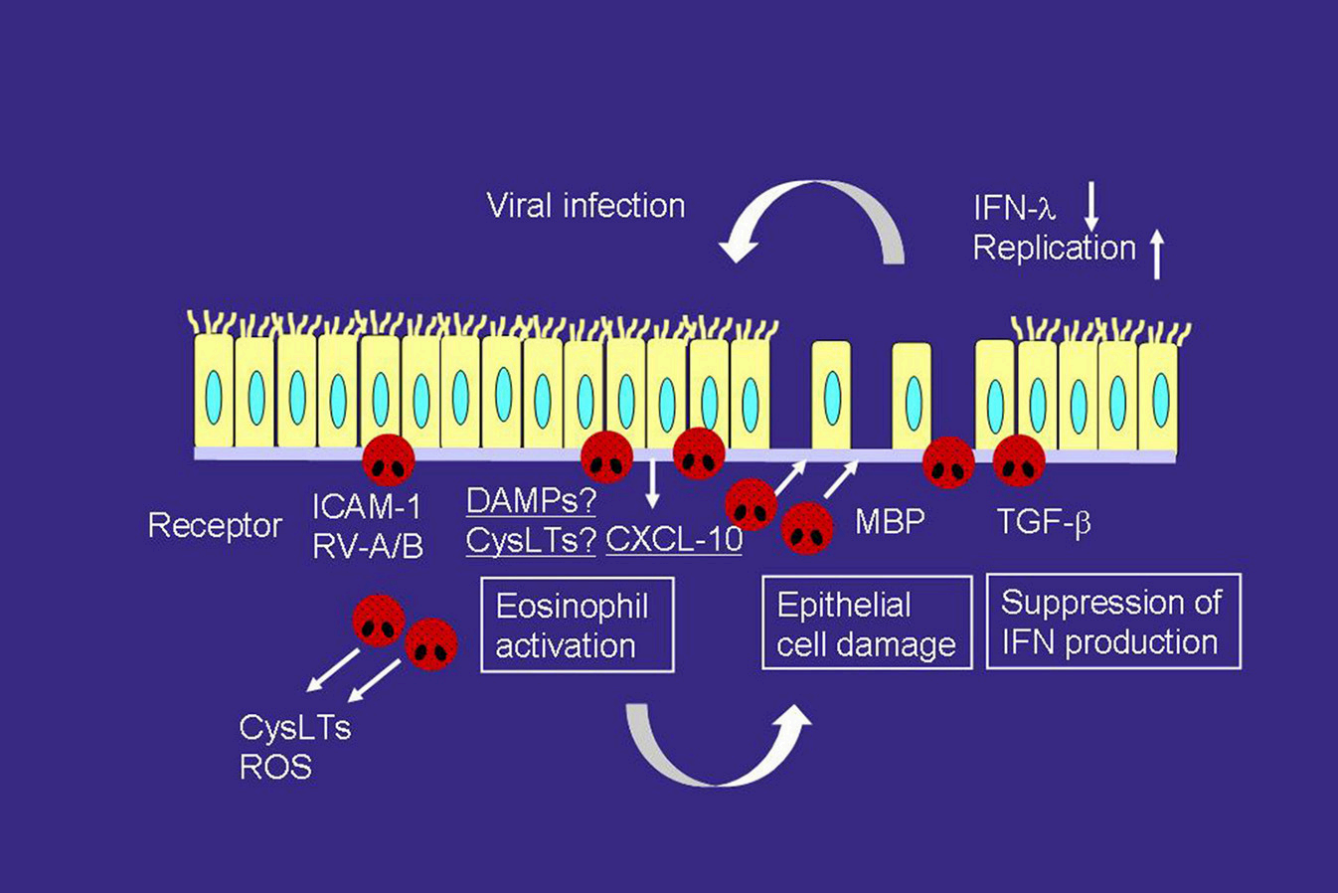
Interactions of viral infection and eosinophils in the development of asthma exacerbation. RV infection releases a variety of mediators, including CXCL-10 and cysLTs, which can directly activate eosinophils and induce asthma exacerbation, from airway epithelial cells. RV infection increases the expression of ICAM-1, which also activates eosinophils. On the other hand, activated eosinophils release MBP, which induces epithelial cell damage. Eosinophils also produce TGF-β, which can suppress the expression of IFN-λ, anti-viral cytokines, on epithelial cells. Therefore, eosinophilic airway inflammation can increase susceptibility to RV infection. ROS, reactive oxygen species; TGF, transforming growth factor.

Recent studies have suggested that the presence of eosinophil inflammation may be a risk factor for virus-related asthma exacerbation (56, 57). High fractional exhaled nitric oxide and sputum eosinophils are associated with an increased risk of future virus-induced exacerbations (57). Epithelial cells are damaged by eosinophil-derived granule products, such as MBP (23), and this increases the susceptibility to RV infection (Figure 2) (58). Furthermore, eosinophils can suppress the RV-induced expression of interferons (IFNs), anti-viral cytokines, including IFN-λ from epithelial cells, likely through the production of TGF-β, resulting in an increased quantity of RV (Figure 2) (56). Therefore, reducing the eosinophil count could be a reasonable strategy for suppressing virus-induced asthma exacerbation.

ICAM-1 is a cellular receptor for the majority of RV-A (major) and all of RV-B (59), and RV infection increases ICAM-1 expression on epithelial cells (60). ICAM-1 is also an adhesion molecule, and adhesion of eosinophils to ICAM-1 can activate the functions of eosinophils (28, 29). Therefore, eosinophil adhesion to epithelial cells via ICAM-1 may activate eosinophils during RV-induced asthma exacerbation (Figure 2). Cadherin-related family member 3 (CDHR3) is a recently found receptor for RV-C (61). In this context, a coding single nucleotide polymorphism (SNP) in CDHR3 has been shown to be related to the severe exacerbation of childhood asthma (62). Moreover, this SNP has been reported to increase the expression of the CDHR3 protein on the cell surface (61, 62), resulting in increased RV-C binding and progeny yields (61). Because the cadherin family members are involved in cell adhesion, eosinophil adhesion to CDHR3 may activate eosinophil functions in a manner similar to that as ICAM-1.

CXCL10 may also play a role in the virus-induced asthma exacerbation (Figure 2). RV infection produces CXCL10 from bronchial epithelial cells *in vitro* and *in vivo* (63). Specifically, concentrations of serum CXCL10 are elevated in virus or RV-induced asthma; correlations are reported between higher levels of CXCL10 and disease severity, including airflow limitation (63). CXCL10 induces eosinophil adhesion, O${}_{2}^{-}$ generation, eosinophil-derived neurotoxin release, and cytokine production through CXCR3, expressed on eosinophils, *in vitro* (64). As for other CXCR3 ligands, CXCL9 is involved in severe asthma (65) and produced from epithelial cells by RV infection (66). CXCL9 induces eosinophil adhesion, O${}_{2}^{-}$ generation, and eosinophil-derived neurotoxin release *in vitro* (64), whereas it inhibits eosinophil chemotaxis through a CCR3-dependent mechanism (67, 68).

CysLTs are upregulated in the asthmatic airways during virus or RV-induced exacerbation (69, 70). Respiratory syncytial virus (RSV) induces LTC_4_ synthase expression on bronchial epithelial cells (71). Therefore, cysLTs are likely to be involved in virus-induced eosinophilic inflammation (Figure 2), and LTRA may be useful for virus-induced asthma treatment. The LTRA montelukast suppresses the respiratory symptoms of RSV bronchiolitis (72) as well as the frequency of virus-induced asthma exacerbation (73). Moreover, montelukast inhibits eosinophil adhesion induced by CXCL10 and ICAM-1 *in vitro* (74), both are virus-infection-related proteins.

Innate immune responses play roles in the development of eosinophilic airway inflammation; this process involves type 2 innate lymphoid cells (ILC2) as well as epithelial cell-related cytokines including IL-33, IL-25, and thymic stromal lymphopoietin, (75, 76). The ILC2 stimulated by these cytokines produce IL-5 and IL-13 and induce eosinophilic inflammation. In fact, ILC2 are upregulated in severe asthmatics (77). Recent studies have suggested that innate immune responses contribute to virus-induced asthma exacerbation. For example, IL-33-dependent type 2 inflammation plays an important role in RV-induced asthma exacerbation *in vivo* (78).

During viral infections, damage-associated molecular pattern molecules (DAMPs) can be released by stressed or damaged cells, and function as endogenous danger signals (79). Damaged epithelial cells are capable of inducing eosinophilic migration, *specific granule protein release*, and cytokine production, likely via the release of DAMPs (80). Uric acid (UA) or adenosine triphosphate (ATP), an important DAMP, activates eosinophil functions *in vitro* (81, 82); however, the role of DAMPs in the development of asthma exacerbation remains unclear.

## Interactions of neutrophils and eosinophils in the development of severe asthma or asthma exacerbation

Both neutrophilic and eosinophilic inflammation may play roles in severe asthma [(83–85)]. Neutrophilic inflammation has been shown to be involved in the pathogenesis of asthma exacerbation (86), which occurs frequently in severe asthma. The European Network for Understanding Mechanisms of Severe Asthma (ENFUMOSA) study suggested that compared with patients with mild-to-moderate asthma, those with severe asthma have both a greater sputum neutrophil count and an increased release of eosinophil-derived mediators (84). IL-8 plays an important role in the accumulation of neutrophils in inflammation sites, and IL-8 expression is upregulated in the airways of severe asthmatic patients (87, 88). In addition, we reported that neutrophils that had migrated to IL-8 induce the transbasement membrane migration of eosinophils *in vitro*, even without eosinophil chemoattractants (89); this neutrophil-induced eosinophil migration is suppressed by LTB_4_ antagonist or platelet-activating factor (PAF) antagonist. LTB_4_ and PAF are potent chemotactic factors for eosinophils (90, 91); therefore, IL-8-stimulated neutrophils can lead to eosinophil accumulation in asthmatic airways through LTB_4_ or PAF (92).

Lipopolysaccharide (LPS) may play a role in inducing IL-8 or neutrophilic inflammation in the airway of severe asthmatics. In the BAL fluid of asthmatic children, LPS levels correlate with airway neutrophils or IL-8 (93). Furthermore, the BAL fluid LPS and genes associated with LPS signaling activation are higher in corticosteroid-resistant asthma (94). Furthermore, a positive correlation is observed between IL-8 mRNA expression in BAL cells and the amount of LPS in BAL fluid (94). Several studies have suggested that Gram-negative bacteria or house dust plays a role in the LPS upregulation in the airways of severe asthmatics. We previously reported that LPS-stimulated neutrophils induce the transbasement membrane migration of eosinophils *in vitro* (95).

IL-17 is another candidate for the upregulation of IL-8 expression (96). Sputum IL-17 concentration correlates with the clinical severity of asthma (97), and the airway expression of IL-17 is increased in severe asthmatics only (98). Furthermore, a correlation between the number of bronchial cells that produce IL-17 and the number of bronchial neutrophils and frequency of asthma exacerbation has been reported (86). In addition, we reported that the dopamine D1-like receptor antagonist attenuates the Th17-mediated immune response and neutrophilic airway inflammation in mice (99) this could be reasonable strategy for controlling neutrophilic airway inflammation in severe asthma or asthma exacerbation.

The role of neutrophil extracellular traps (NETs) in the pathogenesis of asthma exacerbation has recently been highlighted. In a mouse model, RV infection triggered a double-stranded DNA (dsDNA) release that was associated with the formation of NETs; this is known as NETosis (100). Furthermore, in humans, a significant correlation is identified between the release of host dsDNA after RV infection and the exacerbation of type-2 allergic inflammation (100).

## Role of mast cells and prostaglandin (PG) D_2_ in eosinophilic inflammation of asthma

Mast cells also play roles in the development of severe asthma. Mast cell numbers and PGD_2_ concentrations are increased in the lower airway of patients with severe asthma (101, 102). Mast cells are major cellular sources of PGD_2_, and D prostanoid (DP) and chemoattractant receptor-homologous molecule expressed on Th2 cells (CRTH2) are receptors for PGD_2_ (103). Recently, the role of CRTH2 in the pathogenesis of asthma has been highlighted. CRTH2 is expressed on Th2 cells, ILC2, eosinophils, and basophils (103). PGD_2_ induces chemotaxis in eosinophils through CRTH2 (104), and CRTH2 antagonist suppresses eosinophil chemotaxis and respiratory burst (105). CRTH2 antagonists are already being developed (103, 106), and clinical trial data suggest that CRTH2 antagonists may target eosinophilic asthma (103, 107).

## Conclusion

Eosinophils are likely to contribute to the development of asthma exacerbation. This process can involve cytokines, such as IL-5 or GM-CSF, chemokines, such as CCR3 ligands, matricellular proteins, a danger signal, and other cells, such as neutrophils or mast cells.

## Author contributions

All authors listed have made a substantial, direct and intellectual contribution to the work, and approved it for publication.

### Conflict of interest statement

The authors declare that the research was conducted in the absence of any commercial or financial relationships that could be construed as a potential conflict of interest.
